# Validation of the Refugee Health Screener-15 for the assessment of perinatal depression among Karen and Burmese women on the Thai-Myanmar border

**DOI:** 10.1371/journal.pone.0197403

**Published:** 2018-05-21

**Authors:** Gracia Fellmeth, Emma Plugge, Mina Fazel, Prakaykaew Charunwattana, François Nosten, Raymond Fitzpatrick, Julie A. Simpson, Rose McGready

**Affiliations:** 1 Shoklo Malaria Research Unit, Mahidol-Oxford Tropical Medicine Research Unit, Faculty of Tropical Medicine, Mahidol University, Mae Sot, Thailand; 2 Nuffield Department of Population Health, University of Oxford, Oxford, United Kingdom; 3 Centre for Tropical Medicine and Global Health, Nuffield Department of Clinical Medicine, University of Oxford, Oxford, United Kingdom; 4 Department of Psychiatry, University of Oxford, Oxford, United Kingdom; 5 Mahidol-Oxford Tropical Medicine Research Unit, Faculty of Tropical Medicine, Mahidol University, Bangkok, Thailand; 6 Centre for Epidemiology and Biostatistics, Melbourne School of Population and Global Health, University of Melbourne, Melbourne, Victoria, Australia; Tulane University School of Public Health and Tropical Medicine, UNITED STATES

## Abstract

Perinatal depression is common, and left untreated can have significant and long-lasting consequences for women, their children and their families. Migrant women are at particular risk of perinatal depression as a result of a multitude of stressors experienced before, during and after migration. Identification of perinatal depression among migrant women—particularly those living in low- and middle-income regions—remains challenging, partly due to the lack of locally-validated and culturally appropriate screens tools. This study formally validates Burmese and Sgaw Karen versions of the *Refugee Health Screener-15* (RHS-15) as a screening tool for perinatal depression among migrant women living on the Thai-Myanmar border. The *Structured Clinical Interview for the Diagnosis of DSM-IV Disorders* (SCID) was used as the gold-standard comparator. Complete results were obtained for 235 Burmese-speaking and 275 Sgaw Karen-speaking women. Despite displaying reasonable psychometric properties, a number of shortcomings associated with the RHS-15 limited its utility in this setting. The Likert-type response categories of the RHS-15 proved problematic in this low-literacy population. Combined with the relative superiority and greater ease of administration of the SCID, the RHS-15 is not recommended as the tool of choice for detecting perinatal depression in this setting.

## Introduction

Maternal mental health is an important global health challenge and a cornerstone to reducing global inequalities. Perinatal mental disorders are common, and the global burden falls disproportionately upon low- and middle-income countries (LMIC), where an estimated 16% of pregnant and post-partum women experience depression [1–3]. Untreated perinatal depression can have long-lasting consequences for women, and children of depressed mothers are at risk of physical, behavioral and emotional impairments not only in infancy but throughout early childhood, potentially persisting into adolescence [3–5].

Migrant women face a multitude of stressors which place them at increased risk of mental disorders [6–8]. Risk factors work at the individual, family, community and wider societal level and include marginalized status, exposure to traumatic events including sexual and domestic violence, adverse socio-economic circumstances, language barriers and lack of social support in destination countries and poor access to health services. Migrant women who are low-skilled and have resettled within LMIC may constitute a particularly vulnerable subgroup, facing additional challenges such as limited literacy and more severe socio-economic deprivations for themselves and their children [9].

Better identification of affected individuals is key to promoting mental health. Culturally valid tools are essential for detecting illness, quantifying the disease burden, targeting care and monitoring treatment response [10]. For many women, pregnancy is a time of increased contact with health providers and thus offers a valuable screening opportunity. Screening tools allow rapid assessments to be made by non-specialists—an important consideration in LMIC settings where mental health services are commonly lacking or over-stretched. As psychometric properties vary across settings, screening tools must be validated locally prior to use [10]. In addition, screening tools must be considered acceptable and easy to use by the local population in order to be implemented [11].

A previous study of over 600 migrant women conducted on the Thailand-Myanmar border between February 2014 and April 2015 found that the Edinburgh Postnatal Depression Scale (EPDS), one of the most widely-used screening tools for perinatal depression, had poor acceptability among local staff and patients [12]. Women in this deprived and low-literacy setting found the EPDS language difficult, were unfamiliar with Likert-type response categories and found the subtleties between response categories challenging [12]. A number of women were unable to complete the questionnaire and several became distressed. It was necessary, therefore, to find an alternative tool to identify women at risk of depression in this population. The current validation study formed part of a larger study of perinatal mental health on the Thai-Myanmar border [13] and aimed to determine the validity and acceptability of Sgaw Karen and Burmese language versions of the Refugee Health Screener (RHS-15) in this setting. In this paper, the term ‘migrant’ is used to describe any person who has moved from their habitual place of residence, regardless of the circumstances [14]. In this setting, migrant populations include both labour migrants and refugees.

## Methods

### Setting

The Thailand-Myanmar border is home to an estimated 200,000 labour migrants and 145,000 refugees fleeing decades of conflict, poverty and lack of opportunity in Myanmar [15, 16]. Labour migrants live in villages on both sides of the border working in agriculture, manufacturing and the service industry in an area of widespread socioeconomic deprivation. Access to healthcare and education for the majority of labour migrants and their families is limited by their undocumented status. Those that have been granted refugee status live in established camps on the Thai side of the border, the largest of which is Maela with a population of 38,000. Refugees have greater access to healthcare, education and housing as compared with the other migrant groups because non-governmental organisations (NGOs) recognized by the United Nations High Commissioner for Refugees (UNHCR) and the Thailand Ministry of Interior, work within the camps to provide these services.

**Fig 1:**
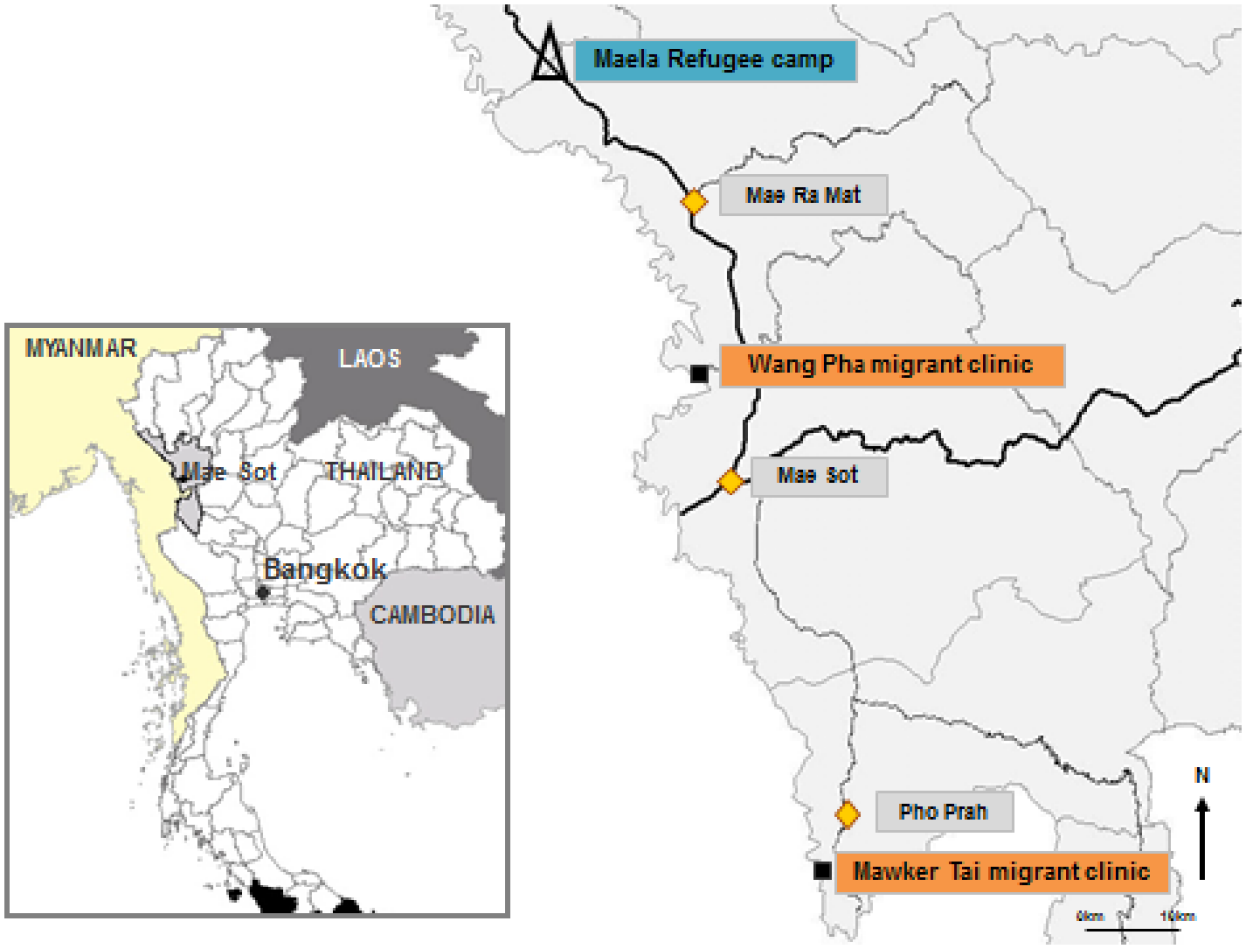
Map of study area showing Shoklo Malaria Research Unit clinic sites. Reprinted from Shoklo Malaria Research Unit under a CC BY license, with permission from the Shoklo Malaria Research Unit, original copyright 2014. Refugee (Δ) and migrant clinics (■).

Migrants in this region constitute a heterogeneous group of Karen, Burman and Burman Muslim ethnicities, each with their own languages, traditions and religious backgrounds. Sgaw Karen, the language of one of Myanmar’s largest ethnic group, is the most commonly spoken language. A smaller proportion speaks Burmese, the official language of Myanmar. The Shoklo Malaria Research Unit (SMRU) has provided maternity care to this border population since 1986. In collaboration with Thailand Public Health, care was initially motivated by the very high rates of maternal mortality due to malaria which were reduced by the antenatal clinic (ANC) services [17]. ANC for rural labour migrant women are located 30-60km north and south of the border town of Mae Sot, Tak Province, at Wang Pha (WPA) and Mawker Tai (MKT) and for refugee women in Maela (MLA) (Fig 1).

### Participants

Participants were first trimester pregnant migrant women attending SMRU antenatal clinics (ANC) at MLA, MKT and WPA. Women were eligible if they were aged 18 years or over, their estimated gestational age (EGA) as determined by ultrasound dating scan was less than 14 weeks, they had a viable pregnancy, planned to deliver at SMRU and were willing and able to participate.

### Ethics

Ethics approval was granted by the University of Oxford Tropical Research Ethics Committee (OxTREC 28–15), Mahidol University Faculty of Tropical Medicine Ethics Committee (TMEC 15–045) and the Tak Border Community Advisory Board (T-CAB 6/2/2015), a committee of local community representatives who assess the acceptability of proposed research [18].

### Instruments

The *Refugee Health Screener-15* (RHS-15) is a fifteen-item screen for symptoms of depression, anxiety and post-traumatic stress disorder (PTSD) developed in conjunction with refugees from Myanmar, Bhutan and Iraq recently resettled in the United States [19, 20]. Items 1–14 ask respondents to rate the frequency of psychological and somatic symptoms on a 5-point Likert scale scored 0 (‘not at all’) to 4 (‘extremely’) and diagrammatically annotated with a beaker filled to varying degrees. Item fifteen is a distress thermometer (DT) which asks respondents to rate their level of distress from 0 (‘no distress’) to 10 (‘extreme distress’). A total score ≥12 on items 1–14 and/or a score of ≥5 on the DT are considered to be a positive screen requiring further assessment. Burmese and Sgaw Karen translations were obtained from the RHS-15 authors [20]. The RHS-15 has not previously been used in Burmese or Karen communities outside of the United States, and there was therefore a need to validate the questionnaire for our study population. The RHS-15 is freely available to researchers and clinicians upon request from *Pathways to Wellness*, rendering it an affordable and sustainable tool to use in low-resource settings [20].

We used the *Structured Clinical Interview for the Diagnosis of DSM-IV Disorders* (SCID) as a diagnostic tool against which to validate the RHS-15 [21]. The ten items relating to depression including low mood, anhedonia, changes in appetite and sleeping, restlessness, energy levels, worthlessness, recurrent thoughts of death, and coping with everyday tasks were selected [21]. Items were translated from English into Burmese and Sgaw Karen by two senior midwives experienced in conducting clinical work and research in the local population and fluent in English, Burmese and Sgaw Karen. Back-translations were conducted by a senior midwife and a physician who had not seen the original English version. Original and back-translated versions were assessed by an English-speaking physician who confirmed semantic equivalence between the two versions. Due to the long-term scarcity of mental health infrastructure in this resource-constrained region, no psychiatrist was available to conduct the translations [22–24]. We used the *Diagnostic and Statistical Manual of Mental Disorders*, *4*^*th*^ *edition* (DSM-IV) criteria to diagnose major and minor depressive disorder [25]. To account for pregnancy status, scoring criteria for sleep and appetite items were considered positive only if they were unrelated to pregnancy status: for example, poor appetite was coded positive if caused by feelings of sadness, and negative if caused by morning sickness.

### Staff and training

The study team consisted of an English-speaking physician (GF), four counsellors and two senior midwives. The physician underwent training by the American Psychiatry Association in conducting the SCID prior to the study. Counsellors and midwives were fluent in Burmese, Sgaw Karen and English. SMRU counsellors and midwives are experienced in working with the local population and are themselves members of the migrant community. Study staff received training in counselling methods and in administering the RHS-15 and SCID. The study was rolled out stepwise across the three sites, enabling the physician to be present for all interviews during the first weeks of enrolment at each site. The physician continued to be available to all sites throughout the study.

### Recruitment

Eligible women were approached by a member of the study team in ANC waiting areas at each of the three sites and provided with verbal and written information about the study. It was explained that participation was voluntary, that non-participation would not affect care and that consent could be withdrawn at any time. Women were able to ask questions before deciding to participate. Those who agreed provided written informed consent by signature or thumbprint. Recruitment ran from October 2015 to April 2016.

### Procedure

Questionnaires were administered by study staff in a private room in Sgaw Karen or Burmese according to women’s preference. Participants first completed a brief demographic questionnaire. The RHS-15 was administered verbally by staff reading the items to participants and then recording responses. This method of verbal administration was used due to low literacy rates within this population and limited comprehension of health-related written information, even among those able to read [26]. Verbal administration is considered acceptable by RHS-15 authors and has been used previously in this setting and elsewhere [19, 27]. After the RHS-15, women completed the SCID. At MKT, the RHS-15 and SCID were conducted by different members of the study team, each of whom was blinded to the results of the other. At MLA and WPA, the RHS-15 and SCID were administered consecutively by the same study team member due to staffing constraints.

All SCID responses were independently scored by the study physician (GF) and an independent physician. Scoring involved using the DSM-IV criteria to give each participant a diagnosis of depression. We included minor as well as major depressive disorder. This decision was based on clinical judgment, as we found at an early stage in the study that the majority of women with minor depressive disorder had symptoms severe enough to warrant being offered treatment. Clinically, these individuals were managed in the same way as those with major depressive disorder. We therefore felt that the combined categories of major and minor depressive disorders provided a more accurate reflection of depression in this setting. Discrepancies in diagnoses were resolved by discussion with a psychiatrist (MF). Women with depression were offered counselling and, when appropriate, anti-depressant medication and followed-up by a physician. Women with severe symptoms or active suicidal ideation were admitted for observation.

### Sample size

We aimed to recruit a total sample of 200 Sgaw Karen-speaking and 200 Burmese-speaking women. This target sample size was based on validation study guidelines suggesting that 200 participants is considered fair [28].

### Statistical analysis

We estimated the proportion (95% confidence interval) of women who met SCID criteria for major or minor depression. Descriptive statistics of demographic characteristics of Burmese-speaking and Sgaw Karen-speaking women, and of RHS-15 outcomes for with and without depression, were presented and compared using Chi^2^ tests for categorical variables and Mann-Whitney U tests for continuous, non-normally distributed variables. We expected mean RHS-15 scores to be significantly higher in depressed compared to non-depressed women: this “known groups” method supports the construct validity of the RHS-15 [29]. We planned to explore possible assessment bias by comparing the proportion of women with depression according to blinding status of SCID interviewers to RHS-15 scores.

To assess the validity of the RHS we calculated the sensitivity, specificity, positive likelihood ratio (LR+) and negative likelihood ratio (LR-) at each cut-off value of the RHS-15. The LR+ represents the probability of participants with depression scoring positive, divided by the probability of participants without depression scoring positive. The higher the value, the more convincingly the RHS-15 score is able to detect depression. Analyses were conducted separately for each language. We determined reliability of the RHS-15 using Cronbach’s alpha and used Youden’s Index, the value at which [(sensitivity + specificity)– 1] is maximised, to identify the point of optimal balance between sensitivity and specificity. We conducted a Receiver Operating Characteristic (ROC) analysis to calculate the area under the curve (AUC) for the RHS-15 and thereby assess criterion accuracy (the proportion of results correctly identified) and validity [30]. We conducted separate analyses for items 1–14 and the distress thermometer (item 15). Statistical analyses were conducted using STATA/IC version 14.1 [31].

## Results

### Baseline characteristics

**Fig 2:**
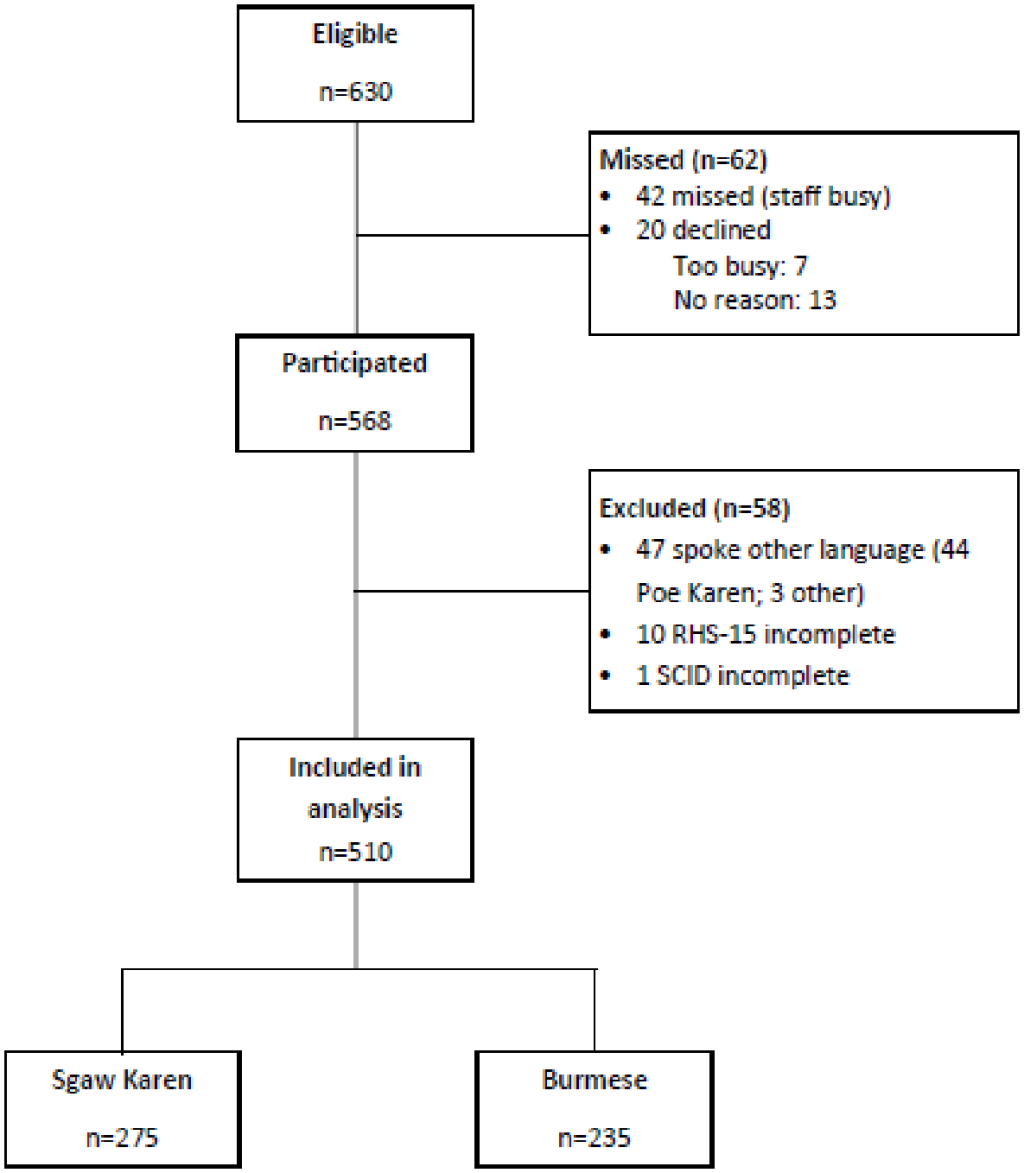
Flow of participants through the study.

Of 630 eligible women who attended ANC during the study period, 569 (90.2%) participated (Fig 2). Women who were eligible but did not participate did not differ significantly from those who participated in terms of age, ethnicity or migrant status. Complete SCID and RHS-15 results were obtained for 235 Burmese-speaking and 275 Sgaw Karen-speaking women. Baseline characteristics of these 510 women included in the current analysis are summarized in Table 1. The prevalence of depression of any severity as diagnosed by the SCID was 7.7% (39/510). Overall, 45.1% (230/510) participants screened positive on the RHS-15 using recommended cut-offs. There were no significant differences between Burmese- and Sgaw Karen-speaking women for depression status or RHS-15 scores. RHS-15 results for women with and without depression are shown in Table 2. Median scores and the proportion of participants scoring positive on the RHS-15 were significantly higher among women with depression compared to women without depression. The prevalence of depression at MKT, where SCID interviewers were blinded to the outcomes of the RHS-15, was lower than at WPA and MLA, where there was no blinding of interviewers (3.3% at MKT vs. 9.5% at WPA and MLA). Because only one site had blinding, and this site also had a lower depression prevalence than the other sites, it was not possible to statistically separate the effects of site and blinding.

**Table 1:** Baseline characteristics of study participants by language of interview.

	Total n = 510	Language of interview		p-value
		Burmese n = 235	Sgaw Karen n = 275	
**Age (years)**				0.332^†^
Median [range]	25 [18–50]	26 [18–50]	25 [18–45]	
**Ethnicity, n (%)**				**<0.001**
Sgaw Karen	269 (52.8)	7 (3.0)	262 (95.3)	
Burman	158 (31.0)	158 (67.2)	0 (0)	
Burman Muslim	43 (8.4)	42 (17.9)	1 (0.4)	
Poe Karen	25 (4.9)	14 (5.96)	11 (4.0)	
Other (e.g. Mon)	15 (2.9)	27 (11.5)	1 (0.4)	
**Religion, n (%)**				**<0.001**
Buddhist	360 (70.6)	181 (77.0)	179 (65.1)	
Christian	106 (20.8)	12 (5.1)	94 (34.2)	
Muslim	44 (8.6)	42 (17.9)	2 (0.7)	
**Literacy, n (%)**				**0.001**
Literate	354 (69.4)	181 (77.0)	173 (62.9)	
**Education**^a^**, n (%)**				**0.002**
<3 years	227 (45.0)	95 (40.8)	132 (48.7)	
3–6 years	150 (29.8)	89 (38.2)	61 (22.5)	
7–10 years	108 (21.4)	41 (17.6)	67 (24.7)	
≥ 10 years	19 (3.8)	8 (3.4)	11 (4.1)	
**Employment**^b^**, n (%)**				**<0.001**
Agriculture	193 (39.8)	126 (57.8)	67 (25.1)	
Selling	42 (8.7)	27 (12.4)	15 (5.6)	
Teacher, health worker	52 (10.7)	7 (3.2)	45 (16.9)	
Other (e.g. factory)	20 (4.1)	9 (4.1)	11 (4.1)	
Unpaid housework	178 (36.7)	49 (22.5)	129 (48.3)	
**Migrant status, n (%)**				**<0.001**
Labour migrant (vs. refugee)	291 (57.1)	185 (78.7)	106 (38.6)	
**Years at current address**^c^				**0.001**
Median [range]	9 [1–39]	8 [1–39]	10 [1–31]	
Proportion ≤ 1 year, n (%)	61 (15.1)	43 (23.8)	18 (8.1)	**<0.001**
**Household size**				
Median [range]	4 [1–14]	3 [1–13]	5 [1–14]	**<0.001**^†^
**Current substance use, n (%)**				
Smoker	52 (10.2)	12 (5.1)	40 (14.6)	**<0.001**
Alcohol	21 (4.1)	6 (2.6)	15 (5.5)	0.100
Betel nut	219 (42.9)	71 (30.2)	148 (53.8)	**<0.001**
**Depression (SCID), n (%)**				
Depression	39 (7.7)	22 (9.4)	17 (6.2)	0.178
**Refugee Health Screener scores**				
*Items 1–14*				
Positive, n (%)	188 (36.9)	91 (38.7)	97 (35.3)	0.421
Median [range]	10 [0–49]	10 [0–49]	9 [0–38]	0.1641^†^
*Distress thermometer*				
Positive, n (%)	125 (24.9)	60 (25.9)	65 (24.1)	0.644
Median [range]	2 [0–10]	3 [0–10]	2 [0–10]	0.1085^†^
*Overall*				
Positive, n (%)	230 (45.1)	114 (48.5)	116 (42.2)	0.152

^†^p-values calculated using Mann-Whitney U test.

All other p-values calculated using Chi-squared test.

Bold denotes statistical significance at p<0.005 level.

^a^n = 6 observations missing.

^b^n = 25 observations missing.

^c^n = 106 observations missing.

**Table 2:** Refugee Health Screener results for participating women according to depression status.

	Depression status on SCID		
	Depression (n = 39)	No depression (n = 463)	p-value
*Items 1–14*			
Positive, n (%)	34 (87.2)	154 (32.7)	**<0.001**
Median [range]	19 [8–49]	9 [0–40]	**<0.001**^†^
*Distress thermometer*			
Positive, n (%)	20 (51.3)	105 (22.7)	**<0.001**
Median [range]	5 [0–10]	2 [0–10]	**<0.001**^†^
*Overall*			
Positive	35 (89.7)	195 (41.4)	**<0.001**

^†^ p-values calculated using Mann-Whitney U test.

All other p-values calculated using Chi-squared test.

Bold denotes statistical significance at p<0.005 level.

### Psychometric properties of the Burmese RHS-15

**Fig 3:**
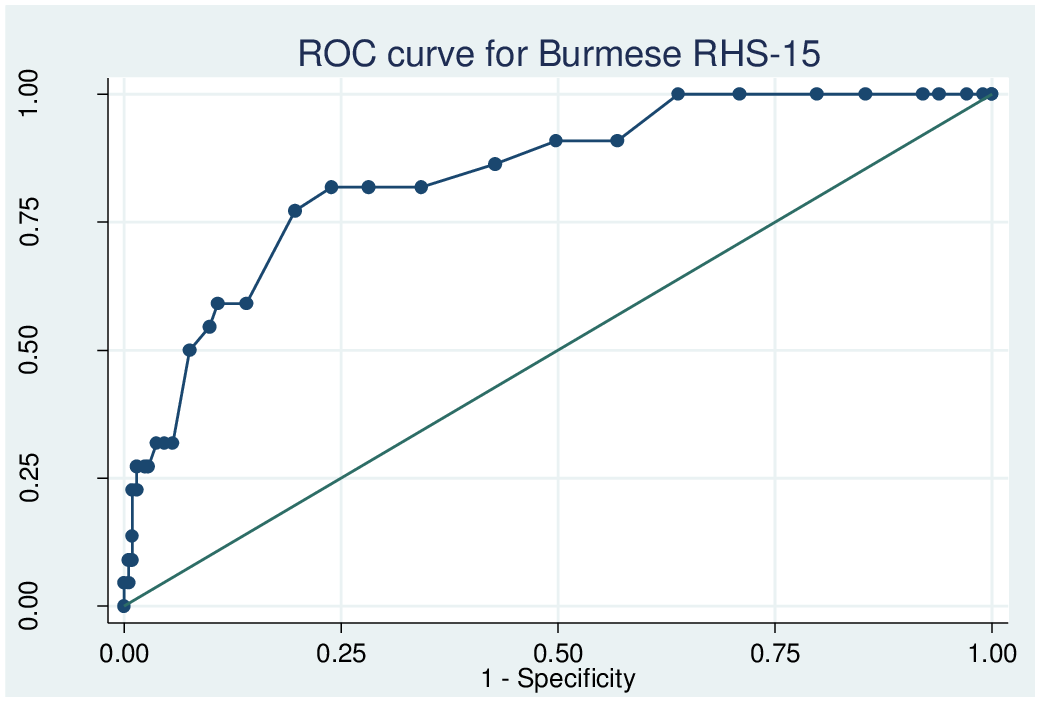
Receiver operating characteristic curve for the Burmese Refugee Health Screener items 1–14.

**Fig 4:**
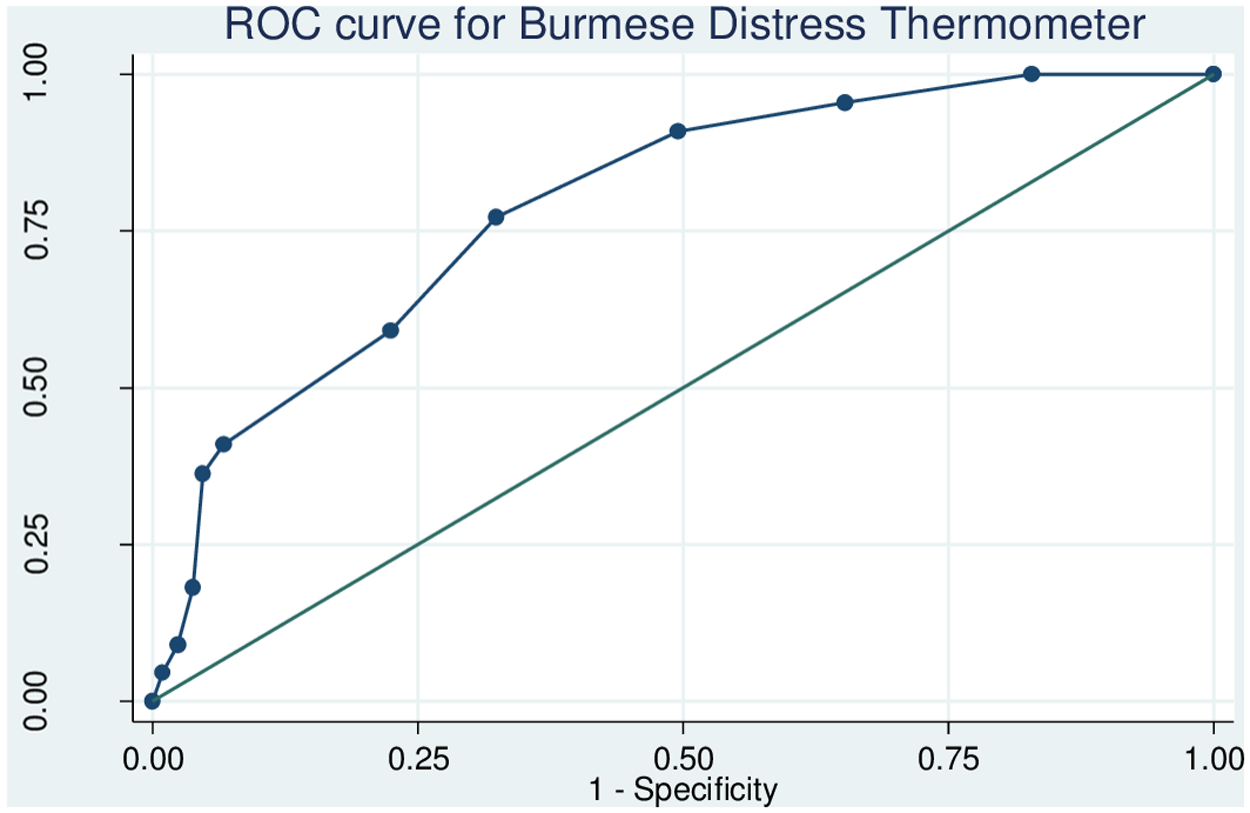
Receiver operating characteristic curve for the Burmese Refugee Health Screener distress thermometer.

The reliability of the Burmese RHS-15 as determined using Cronbach’s alpha was 0.63. Omitting unhelpful individual items of the RHS-15 did not improve the reliability significantly producing a range of alpha values between 0.56 and 0.66. The sensitivity, specificity and likelihood ratios for each cut-off of items 1–14 of the Burmese RHS-15 are shown in S1 Table. The ROC curve for items 1–14 is shown in Fig 3. The area under the curve was 0.84 (95% CI 0.76–0.93). Youden’s index for items 1–14 was maximized (0.58) at a cut-off of ≥14, suggesting that this is the optimal threshold for our population. At this cut-off, the sensitivity of items 1–14 was 81.8% (95% CI 61.5–92.7%), specificity was 76.1% (95% CI 69.9–81.3%), LR+ was 3.42, LR- was 0.24 and the proportion correctly identified was 76.6%. The sensitivity, specificity and likelihood ratios for each cut-off of the distress thermometer are shown in S2 Table. The ROC curve for the distress thermometer is shown in Fig 4. The area under the curve for the distress thermometer was 0.79 (95% CI 0.70–0.88), and Youden’s index was maximized (0.45) at a threshold of ≥4. At this optimal cut-off, sensitivity of the distress thermometer was 77.3% (95% CI 56.5–89.9%), specificity was 66.7% (95% CI 60.1–72.7%), LR+ was 2.39, LR- was 0.34 and the proportion correctly identified was 68.5%. Using the optimal thresholds of items 1–14 ≥14 and distress thermometer score ≥4, 47.7% (112/235) of women screened positive.

### Psychometric properties of the Sgaw Karen RHS-15

**Fig 5:**
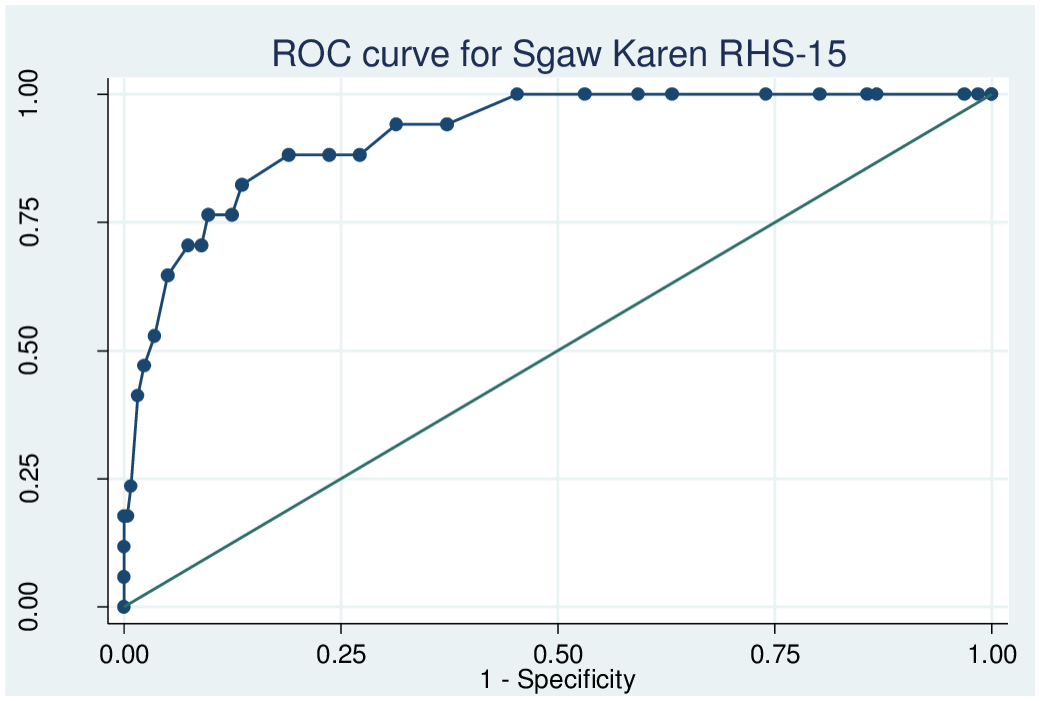
Receiver operating characteristic curve for the Sgaw Karen Refugee Health Screener items 1–14.

**Fig 6:**
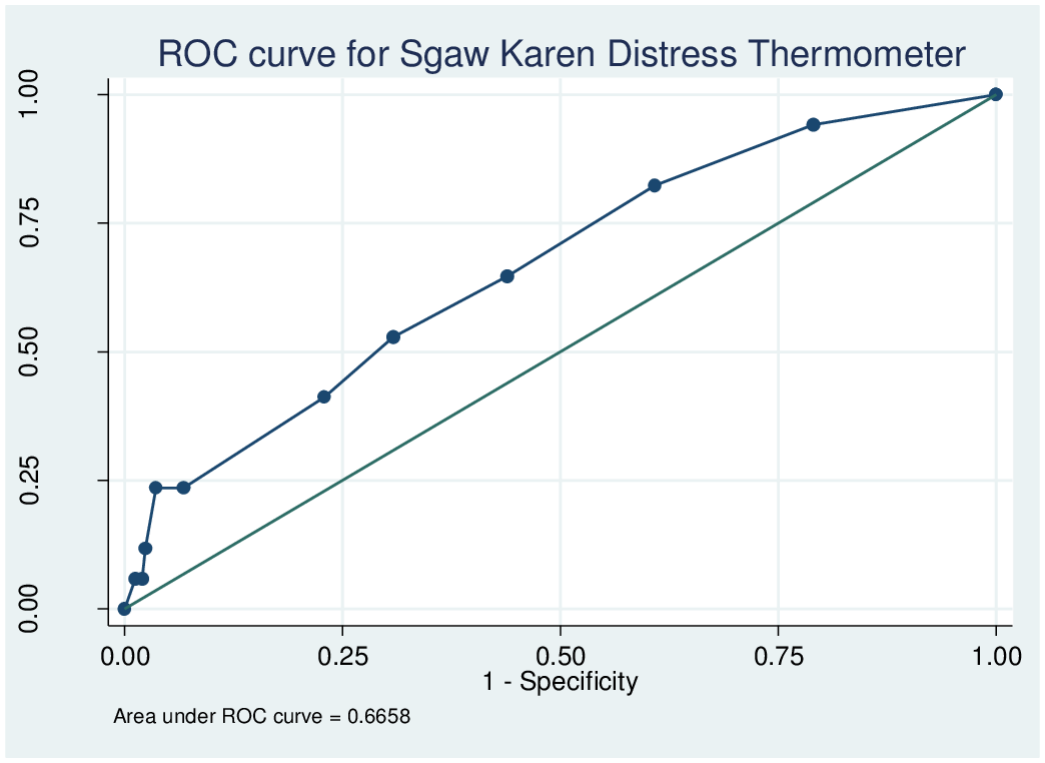
Receiver operating characteristic curve for the Sgaw Karen Refugee Health Screener distress thermometer.

The reliability of the Sgaw Karen RHS-15 as determined using Cronbach’s alpha was 0.56, with values ranging between 0.48 and 0.52 when individual items were omitted. The sensitivity, specificity and likelihood ratios for each cut-off of items 1–14 of the Sgaw Karen RHS-15 are shown in S3 Table. The ROC curve for items 1–14 is shown in Fig 5. The area under the curve for items 1–14 was 0.92 (95% CI 0.86–0.98), and Youden’s index was maximized (0.692) at a threshold of ≥15. At this cut-off, the sensitivity of items 1–14 was 88.2% (95% CI 65.7–96.7%), specificity was 81.0% (95% CI 75.8–85.3%), LR+ was 4.65, LR- was 0.15 and 81.5% of women were correctly identified. The sensitivity, specificity and likelihood ratios for the Sgaw Karen distress thermometer are shown in S4 Table. The ROC curve for the distress thermometer is shown in Fig 6. The area under the curve for the distress thermometer was 0.67 (95% CI 0.53–0.78), and Youden’s index was maximized (0.22) at a threshold of ≥4. At this cut-off, the sensitivity of the distress thermometer was 52.9% (95% CI 31.0–73.8%), specificity was 69.2% (95% CI 63.2–74.5%), LR+ was 1.72, LR- was 0.68 and the proportion correctly identified was 68.2%. Using the optimal thresholds of items 1–14 ≥15 and distress thermometer score ≥4, 41.1% (113/275) of women screened positive.

## Discussion

This study examines the validity of the Burmese and Sgaw Karen RHS-15 as a screening tool for perinatal depression in women on the Thai-Myanmar border. The combined prevalence of major and minor depression during the first trimester of pregnancy as assessed using the SCID was 7.7%. This is line with estimates reported in a systematic review of refugees resettled in Western countries, which found a pooled prevalence of major depression of 5% [32]. Overall, the RHS-15 performed adequately, displaying reasonable sensitivity and specificity in both Burmese and Sgaw Karen languages. However, there were also a number of shortcomings which limited the utility of the RHS-15 in this setting.

On the Burmese RHS-15, the optimal cut-off for items 1–14 was ≥14, yielding good sensitivity and specificity at 81.8% and 76.1%, respectively. The distress thermometer in Burmese performed slightly less well with a sensitivity of 77.3% and specificity of 67.6% at the optimal cut-off of ≥4. On the Sgaw Karen RHS-15, the optimal cut-off for items 1–14 was ≥15, yielding good sensitivity and specificity at 88.2% and 81.0%, respectively. The distress thermometer in Sgaw Karen performed significantly less well: at the optimal cut-off of ≥4 sensitivity and specificity were low at 52.9% and 69.2%, respectively. Reliability as determined by Cronbach’s alpha was low in both languages (0.63 in Burmese; 0.56 in Sgaw Karen). However, as the RHS-15 assesses for anxiety and post-traumatic stress disorder as well as depression, the low alpha values may reflect the multiple dimensions of the test rather than poor internal consistency of the scale [33].

In the original validation of the RHS-15 among refugees in Washington State, USA, Hollifield *et al*. found significantly higher sensitivity (100%), specificity (91%) and reliability (Cronbach’s alpha 0.92) among Burmese participants [19]. A number of explanations are possible for these differences. Firstly, the current sample of pregnant migrant women in a LMIC differs considerably from the original study’s sample of men and women refugees in the USA. Given that only a small proportion of migrant populations are selected for formal resettlement programmes, the US sample is likely to differ significantly and systematically from general migrant populations prior to resettlement. Secondly, Hollifield *et al*.’s small sample of 50 Burmese participants, of whom only six had a diagnosis of depression, suggests that results must be interpreted with caution. Furthermore Hollifield *et al*.’s group of ‘Burmese’ participants in fact include four distinct ethnicities (Burmese, Karen, Chin and Karenni) and two interview languages (Burmese and Sgaw Karen). Not disaggregating results by language or ethnicity may have masked differences between groups.

A number of important contextualising factors need to be considered when interpreting findings. Firstly, factors relating to language and literacy are likely to have influenced results. Participants found it difficult to select the appropriate Likert-type response category on the RHS-15, and it was often necessary to discuss at length the distinctions between ‘a little bit’, ‘moderately’ and ‘quite a bit’–none of which have a direct translation into Burmese or Sgaw Karen. Previous studies have described the challenges of using Likert scales in low-literacy populations [34–36]. The literacy rate in our sample was 69%, but this was based on participants’ self-reported answers to the question, “Are you able to read and write fluently?”. A previous study in this population assessed literacy objectively by setting participants a short reading test, resulting in lower rates of 47% [26]. The previous study found that not all women who reported being able to read well were in fact able to complete the reading test fluently, and it therefore likely that our self-reported figure of 69% is an over-estimation of current literacy rates [26]. An inability to read has been associated with a loss of discriminant power of five-category response scales such as the RHS-15 scale, and it has been suggested that simpler response scales such as a three-point scale are more reliable in low-literacy populations [34]. Even among those able to read fluently, health literacy—the ability to process and understand health information and make appropriate health decisions—has generally been low on the Thai-Myanmar border setting [37]. It is unclear why the distress thermometer, despite being one of the most readily understood items of the RHS-15, was a poorer predictor of depression status than the more difficult-to-answer items 1–14.

**Fig 7:**
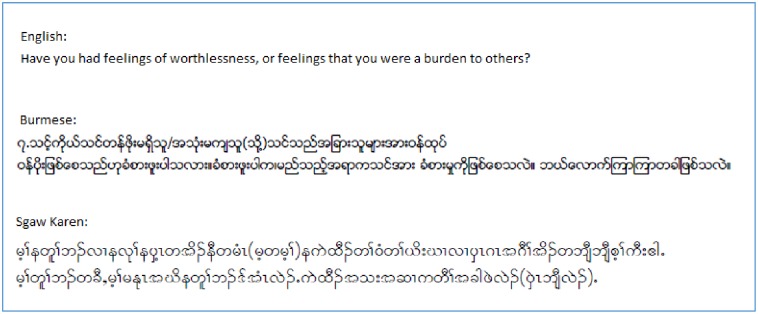
Graphical illustration of sentence lengthening upon translation from English into Burmese and Sgaw Karen languages.

A further constraint may have been the linguistic characteristics of Burmese and Sgaw Karen. As an ethnic language with a strong oral tradition, Sgaw Karen in particular has a limited scope for nuanced questioning and linguistic precision, especially around Western constructs of mental distress and its symptoms. The absence of a Burmese or Sgaw Karen word for depression means that alternative words with similar meanings are needed to convey its meaning. Fig 7 illustrates graphically how a relatively short sentence in English becomes lengthened following translation into Burmese and Sgaw Karen, highlighting the complexity involved in conveying some of the SCID and RHS-15 questions.

A further important consideration arose from the relative superiority of the comparator instrument. It became apparent during the study that in this particular setting, the SCID had a number of strengths over the RHS-15. The SCID proved not only more straight-forward to administer, but also elicited a greater level of detail and depth than the RHS-15. The open questions format of the SCID allowed women to describe their feelings in their own words, and often, valuable additional information around their personal and social circumstances emerged which helped to contextualise their psychological state. The SCID therefore added a breadth and depth to the clinical history that far exceeded what was elicited by the RHS-15. Importantly, on average, the SCID saved time. For women with no symptoms of depression, the SCID took less time to administer than the RHS-15. For women with symptoms, the SCID took longer to administer than the RHS-15, butremained faster than the alternative of administering the RHS-15 first followed by the SCID, as would be required by a screen-positive RHS-15.

Added to these two contextual factors came the fact that despite good sensitivity and specificity of certain aspects of the RHS-15, a number of its properties were sub-optimal. The fact that overall, 44% (225/510) of women screened positive on the RHS-15 using the optimal thresholds for our population is problematic given that each of these screen-positive women requires further assessment in the form of a diagnostic interview. Follow-up of almost one in every two women attending ANC is unfeasible in a resource-constrained setting such as ours with numerous competing health priorities. This limitation of the RHS-15, along with women’s difficulty with the Likert-type scale and the relative superiority of the SCID, led to the decision not to continue using the RHS-15 in our setting.

The greatest strength of our study is that, to our knowledge, it is the first to use the RHS-15 in a LMIC setting. The majority of today’s migration flows occur within LMIC but despite this, research on migrants in LMIC is severely lacking [9]. Understanding and addressing the needs of this group is paramount, particularly as their experiences and circumstances are likely to differ significantly from migrants who resettle in higher-income settings, who represent a minority of the total population of migrants globally. A further strength is our sample size of 510 which is impressive given the high rates of mobility in this population. Our sample represented 81% of all eligible women. This high response rate suggests that our results are representative of the wider migrant community served by SMRU.

There were a number of limitations to our study. Firstly, gold standard assessments of depression were made by non-specialists. The SCID was designed to be administered by a clinician or trained mental health professional, rather than by minimally-trained healthcare workers. The lack of mental health expertise in our setting meant that specialist involvement was not an option available to us, and this is likely to be the case in other LMIC settings [23, 24]. However, we maximised accuracy by using trained clinicians with extensive experience of working within this community. Arguably, local staff might in fact be better placed to understand and respond to culturally-specific presentations of mental disorders than less locally-experienced experts in mental health [11]. In addition, it would neither be desirable nor sustainable in our setting for common mental disorders to be diagnosed and managed by specialists [11]. Engaging frontline, local staff to conduct this study provided experience and expertise within the community and ensured that screening procedures and management patterns would be sustainable in the long-run. It is important to note that although the SCID served as a useful tool in our setting, the high levels of training ideally required to achieve optimal SCID results is likely to be unfeasible in many other resource-poor settings. The applicability and appropriateness of different tools for the assessment of depression across cultures and settings is highly context-specific, and findings of what worked in our particular setting may not necessary be applicable elsewhere.

A second limitation was that due to staffing constraints, SCID assessors were not blinded to RHS-15 scores at two out of the three study sites. Because blinding was only possible at one site, and because this site also had a lower prevalence of depression, it was not possible to explore whether there was an association with blinding status and depression. We are therefore unable to say whether or not blinding had a significant effect on the diagnosis of depression using the SCID. Thirdly, the fact that the RHS-15 was verbally-administered by interviewers rather than self-completed may have affected results. Face-to-face, interviewer-administered questions may be prone to social desirability bias and a lower willingness to disclose sensitive information [38]. However, this method, which places the least possible burden on participants, was necessary in our low-literacy setting [38]. There may in fact have been advantages to our approach: a friendly and sensitive interviewer can encourage the disclosure of information as well as provide or seek clarification when necessary [39], and the fact that interviewers were local staff who are themselves part of the migrant community may have helped to establish trust. During the consent process, we ensured women understood that any information they disclosed would be confidential.

Finally, the gold standard SCID has not itself been validated in this setting. The SCID remains one of the most widely used diagnostic instruments globally and was selected based on the lack of any alternative, locally-developed tools. The possibility that culturally-specific symptoms of depression were missed cannot be ruled out. Somatic symptoms, for example, are common in non-Western cultures but may not have been picked up by the SCID which focuses on psychological symptoms [1, 40]. In a previous study exploring pregnant women’s perceptions of mental illness on the Thai-Myanmar border, women included a ‘heavy head’, tingling and numbness as characteristics of depression [41]. Women who presented with only these symptoms may not have met the DSM-IV criteria and this could have led to an under-estimation of perinatal depression prevalence. However, the open questions format allowed an array of symptoms to be volunteered by participants, and these were followed-up by staff accordingly.

## Conclusion

As is typical of other resource-constrained settings, mental health services are severely lacking on the Thai-Myanmar border. Mental disorders during pregnancy and the post-partum period have immediate and long-term consequences for mothers, children and wider society. The lack of appropriate, locally validated screening tools limit the ability to identify and monitor disorders such as perinatal depression, perpetuating its low priority on the global agenda for marginalised and vulnerable populations. Although the RHS-15 demonstrated good sensitivity and specificity on the Thai-Myanmar border, the SCID was able to elicit more detailed and culturally-relevant information in the same amount of time, leading us to choose the SCID as the tool of choice in our setting. Further research is required on the role of health literacy in mental health screening. With the majority of global migration flows occurring within LMIC regions, research from these regions is an urgent research priority. As long as the needs of the most vulnerable communities remain under-researched, these needs will also be remain insufficiently addressed.

## Supporting information

S1 Table Sensitivity, specificity and likelihood ratios for each cut-off of items 1–14 of the Burmese RHS-15. (DOCX)

S2 Table Sensitivity, specificity and likelihood ratios for each cut-off of the distress thermometer (item 15) of the Burmese RHS-15. (DOCX)

S3 Table Sensitivity, specificity and likelihood ratios for each cut-off of items 1–14 of the Sgaw Karen RHS-15. (DOCX)

S4 Table Sensitivity, specificity and likelihood ratios for each cut-off of the distress thermometer (item 15) of the Sgaw Karen RHS-15. (DOCX)

S1 Text Application form for datasets under the custodianship of Mahidol Oxford Tropical Medicine Research Unit (MORU) Tropical Network. (DOCX)

S2 Text Copyright statement for Fig 1. (PDF)

## References

[1] Fisher J, Cabral de Mello M, Patel V, Rahman A, Tran T, Holton S, et al Prevalence and determinants of common perinatal mental disorders in women in low- and lower-middle-income countries: a systematic review. Bulletin of the World Health Organization. 2012;90(2):139g–49g. Epub 2012/03/17. doi: 10.2471/BLT.11.091850 . 22423165 10.2471/BLT.11.091850 PMC3302553

[2] Lancaster CA, Gold KJ, Flynn HA, Yoo H, Marcus SM, Davis MM. Risk factors for depressive symptoms during pregnancy: a systematic review. American journal of obstetrics and gynecology. 2010;202(1):5–14. Epub 2010/01/26. doi: 10.1016/j.ajog.2009.09.007 . 20096252 10.1016/j.ajog.2009.09.007 PMC2919747

[3] Howard LM, Molyneaux E, Dennis CL, Rochat T, Stein A, Milgrom J. Non-psychotic mental disorders in the perinatal period. Lancet (London, England). 2014;384(9956):1775–88. Epub 2014/12/03. doi: 10.1016/s0140-6736(14)61276-9 . 25455248 10.1016/S0140-6736(14)61276-9

[4] Stein A, Pearson RM, Goodman SH, Rapa E, Rahman A, McCallum M, et al Effects of perinatal mental disorders on the fetus and child. Lancet (London, England). 2014;384(9956):1800–19. Epub 2014/12/03. doi: 10.1016/s0140-6736(14)61277-0 . 25455250 10.1016/S0140-6736(14)61277-0

[5] Stewart RC. Maternal depression and infant growth: a review of recent evidence. Maternal & child nutrition. 2007;3(2):94–107. Epub 2007/03/16. doi: 10.1111/j.1740-8709.2007.00088.x . 17355442 10.1111/j.1740-8709.2007.00088.x PMC6860855

[6] Collins CH, Zimmerman C, Howard LM. Refugee, asylum seeker, immigrant women and postnatal depression: rates and risk factors. Archives of women’s mental health. 2011;14(1):3–11. Epub 2010/12/15. doi: 10.1007/s00737-010-0198-7 . 21153849 10.1007/s00737-010-0198-7

[7] Zimmerman C, Kiss L, Hossain M. Migration and health: a framework for 21st century policy-making. PLoS medicine. 2011;8(5):e1001034 Epub 2011/06/02. doi: 10.1371/journal.pmed.1001034 . 21629681 10.1371/journal.pmed.1001034 PMC3101201

[8] Whitmill J, Blanton C, Doraiswamy S, Cornier N, Schilperood M, Spiegel P, et al Retrospective analysis of reproductive health indicators in the United Nations High Commissioner for Refugees post-emergency camps 2007–2013. Conflict and health. 2016;10:3 Epub 2016/03/11. doi: 10.1186/s13031-016-0069-6 . 26962327 10.1186/s13031-016-0069-6 PMC4784418

[9] Fellmeth G, Fazel M, Plugge E. Migration and perinatal mental health in women from low- and middle-income countries: a systematic review and meta-analysis. BJOG: an international journal of obstetrics and gynaecology. 2016 Epub 2016/06/21. doi: 10.1111/1471-0528.14184 . 27320110 10.1111/1471-0528.14184

[10] Ali GC, Ryan G, De Silva MJ. Validated Screening Tools for Common Mental Disorders in Low and Middle Income Countries: A Systematic Review. PloS one. 2016;11(6):e0156939 Epub 2016/06/17. doi: 10.1371/journal.pone.0156939 . 27310297 10.1371/journal.pone.0156939 PMC4911088

[11] Marley JV, Kotz J, Engelke C, Williams M, Stephen D, Coutinho S, et al Validity and Acceptability of Kimberley Mum’s Mood Scale to Screen for Perinatal Anxiety and Depression in Remote Aboriginal Health Care Settings. PloS one. 2017;12(1):e0168969 Epub 2017/01/31. doi: 10.1371/journal.pone.0168969 . 28135275 10.1371/journal.pone.0168969 PMC5279756

[12] Ing H, Fellmeth G, White J, Stein A, Simpson JA, McGready R. Validation of the Edinburgh Postnatal Depression Scale (EPDS) on the Thai-Myanmar border. Tropical Doctor. 2017. 10.1177/0049475517717635 PMC5613805 28699396

[13] Fellmeth G, Plugge EH, Carrara V, Fazel M, Oo MM, Phichitphadungtham Y, et al Migrant perinatal depression study: a prospective cohort study of perinatal depression on the Thai-Myanmar border. BMJ open. 2018;8(1):e017129 Epub 2018/01/08. doi: 10.1136/bmjopen-2017-017129 . 29306876 10.1136/bmjopen-2017-017129 PMC5780720

[14] 25 ed Geneva: International Organization for Migration (IOM); 2011 Glossary on Migration

[15] Pearson R, Kusakabe K. Thailand’s hidden workforce: Burmese migrant women factory workers. London: Zed Books; 2012.

[16] Dunlop N. Invisible people: stories of migrant labourers in Thailand. Bangkok: Raks Thai Foundation; 2011.

[17] McGready R, Boel M, Rijken MJ, Ashley EA, Cho T, Moo O, et al Effect of early detection and treatment on malaria related maternal mortality on the north-western border of Thailand 1986–2010. PloS one. 2012;7(7):e40244 Epub 2012/07/21. doi: 10.1371/journal.pone.0040244 . 22815732 10.1371/journal.pone.0040244 PMC3399834

[18] Maung Lwin K, Cheah PY, Cheah PK, White NJ, Day NP, Nosten F, et al Motivations and perceptions of community advisory boards in the ethics of medical research: the case of the Thai-Myanmar border. BMC medical ethics. 2014;15:12 Epub 2014/02/19. doi: 10.1186/1472-6939-15-12 . 24533875 10.1186/1472-6939-15-12 PMC3929312

[19] Hollifield M, Verbillis-Kolp S, Farmer B, Toolson EC, Woldehaimanot T, Yamazaki J, et al The Refugee Health Screener-15 (RHS-15): development and validation of an instrument for anxiety, depression, and PTSD in refugees. General hospital psychiatry. 2013;35(2):202–9. Epub 2013/01/26. doi: 10.1016/j.genhosppsych.2012.12.002 . 23347455 10.1016/j.genhosppsych.2012.12.002

[20] Pathways to Wellness. Introductory and referral scripts for the RHS-15 Refugee Health Technical Assistance Center (RHTAC): http://refugeehealthta.org/wp-content/uploads/2012/09/RHS15_Packet_PathwaysToWellness-1.pdf 2012 [cited 2017 13 March]. The RHS-15 is a tool for screening refugees for emotional distress and mental health. Packet includes the RHS- tool, background on the tool’s development, and guidelines on using the RHS- with recently resettled refugees. RHS- has also been translated into Somali, Russian, Arabic, Burmese, Karen, and Nepali.]. http://www.lcsnw.org/pathways/index.html.

[21] First MB, Williams JBW, Spitzer RL, Gibbon M. Structured Clinical Interview for DSM-IV-TR Axis 1 Disorders, Clinical Trials version (SCID-CT). New York: New York State Psychiatric Institute; 2007.

[22] Jenkins R, Kydd R, Mullen P, Thomson K, Sculley J, Kuper S, et al International migration of doctors, and its impact on availability of psychiatrists in low and middle income countries. PloS one. 2010;5(2):e9049 Epub 2010/02/09. doi: 10.1371/journal.pone.0009049 . 20140216 10.1371/journal.pone.0009049 PMC2816209

[23] Zaw KM. Psychiatric services in Myanmar: a historical perspective. Psychiatric Bulletin. 1997;21:506–9.

[24] Office WHOWMC, Myanmar MoH. WHO-AIMS Report on Mental Health Systems in Myanmar. Myanmar: World Health Organization, 2006 Contract No.: http://www.who.int/mental_health/evidence/myanmar_who_aims_report.pdf.

[25] American Psychiatry Association. Diagnostic and Statistical Manual of Mental Disorders, fourth edition (DSM-IV). Washington, D.C.: American Psychiatric Association; 2000.

[26] Carrara VI, Hogan C, De Pree C, Nosten F, McGready R. Improved pregnancy outcome in refugees and migrants despite low literacy on the Thai-Burmese border: results of three cross-sectional surveys. BMC pregnancy and childbirth. 2011;11:45 Epub 2011/06/18. doi: 10.1186/1471-2393-11-45 . 21679475 10.1186/1471-2393-11-45 PMC3142536

[27] Johnson-Agbakwu CE, Allen J, Nizigiyimana JF, Ramirez G, Hollifield M. Mental health screening among newly arrived refugees seeking routine obstetric and gynecologic care. Psychological services. 2014;11(4):470–6. Epub 2014/11/11. doi: 10.1037/a0036400 . 25383999 10.1037/a0036400 PMC4228798

[28] Comrey AL, Lee HB. A first course in factor analysis. Hillsdale, New Jersey: Lawrence Erlbaum Associates; 1992.

[29] Devellis RF. Scale development: theory and applications (2nd edition). Newbury Park, California: Sage Publications; 2003.

[30] Hanley JA, McNeil BJ. The meaning and use of the area under a receiver operating characteristic (ROC) curve. Radiology. 1982;143(1):29–36. doi: 10.1148/radiology.143.1.7063747 7063747 10.1148/radiology.143.1.7063747

[31] StataCorp. Stata Statistical Software: Release 14. College Station, Texas: StatCorp LP; 2015.

[32] Fazel M, Wheeler J, Danesh J. Prevalence of serious mental disorder in 7000 refugees resettled in western countries: a systematic review. Lancet (London, England). 2005;365(9467):1309–14. Epub 2005/04/13. doi: 10.1016/s0140-6736(05)61027-6 . 15823380 10.1016/S0140-6736(05)61027-6

[33] Tavakol M, Dennick R. Making sense of Cronbach’s alpha. International Journal of Medical Education. 2011;2:53–5. doi: 10.5116/ijme.4dfb.8dfd 28029643 10.5116/ijme.4dfb.8dfd PMC4205511

[34] Chachamovich E, Fleck MP, Power M. Literacy affected ability to adequately discriminate among categories in multipoint Likert Scales. Journal of clinical epidemiology. 2009;62(1):37–46. Epub 2008/07/16. doi: 10.1016/j.jclinepi.2008.03.002 . 18619806 10.1016/j.jclinepi.2008.03.002

[35] Bernal H, Wooley S, Schensul JJ. The challenge of using Likert-type scales with low-literate ethnic populations. Nursing research. 1997;46(3):179–81. Epub 1997/05/01. . 9176508 10.1097/00006199-199705000-00009

[36] D’Alonzo KT. Evaluation and revision of questionnaires for use among low-literacy immigrant Latinos. Revista latino-americana de enfermagem. 2011;19(5):1255–64. Epub 2011/10/28. . 22030592 10.1590/s0104-11692011000500025 PMC3286231

[37] Karimi S, Keyvanara M, Hosseini M, Jazi MJ, Khorasani E. The relationship between health literacy with health status and healthcare utilization in 18–64 years old people in Isfahan. Journal of education and health promotion. 2014;3:75 Epub 2014/08/01. . 25077168 10.4103/2277-9531.134910 PMC4113988

[38] Bowling A. Mode of questionnaire administration can have serious effects on data quality. Journal of public health (Oxford, England). 2005;27(3):281–91. Epub 2005/05/05. doi: 10.1093/pubmed/fdi031 . 15870099 10.1093/pubmed/fdi031

[39] Cook TL, Shannon PJ, Vinson GA, Letts JP, Dwee E. War trauma and torture experiences reported during public health screening of newly resettled Karen refugees: a qualitative study. BMC international health and human rights. 2015;15:8 Epub 2015/04/17. doi: 10.1186/s12914-015-0046-y . 25881236 10.1186/s12914-015-0046-y PMC4414007

[40] Patel V, Sumathipala A. Psychological approaches to somatisation in developing countries. Advances in Psychiatric Treatment. 2005;12:54–62.

[41] Fellmeth G, Plugge E, Paw MK, Charunwatthana P, Nosten F, McGready R. Pregnant migrant and refugee women’s perceptions of mental illness on the Thai-Myanmar border: a qualitative study. BMC pregnancy and childbirth. 2015;15:93 Epub 2015/04/18. doi: 10.1186/s12884-015-0517-0 . 25884681 10.1186/s12884-015-0517-0 PMC4464696

