# Supplementary material for: Validation of the Refugee Health Screener-15 for the assessment of perinatal depression among Karen and Burmese women on the Thai-Myanmar border
Source: PLoS One. 2018 May 21;13(5):e0197403. doi: 10.1371/journal.pone.0197403 (PMC5962314; doi:10.1371/journal.pone.0197403)
Supplement: S1 Table — (DOCX) [file pone.0197403.s001.docx]

**S1 Table.** Sensitivity, specificity, likelihood ratios and proportion correctly classified using the Burmese RHS-15 items 1-14 (n=235)

| **RHS-15 cut-off** | **Sensitivity** (%) | **Specificity** (%) | **Correctly classified** (%) | **Positive likelihood ratio** | **Negative likelihood ratio** |
| --- | --- | --- | --- | --- | --- |
| ≥0 | 100.0 | 0.0 | 9.4 | 1.00 | - |
| ≥1 | 100.0 | 0.9 | 10.2 | 1.01 | 0.00 |
| ≥2 | 100.0 | 2.8 | 11.9 | 1.03 | 0.00 |
| ≥3 | 100.0 | 6.1 | 14.9 | 1.07 | 0.00 |
| ≥4 | 100.0 | 8.0 | 16.6 | 1.09 | 0.00 |
| ≥5 | 100.0 | 14.6 | 22.6 | 1.17 | 0.00 |
| ≥6 | 100.0 | 20.2 | 27.7 | 1.25 | 0.00 |
| ≥7 | 100.0 | 29.1 | 35.7 | 1.41 | 0.00 |
| ≥8 | 100.0 | 36.2 | 42.1 | 1.57 | 0.00 |
| ≥9 | 90.9 | 43.2 | 47.7 | 1.60 | 0.21 |
| ≥10 | 90.9 | 50.2 | 54.0 | 1.83 | 0.18 |
| ≥11 | 86.4 | 57.3 | 60.0 | 2.02 | 0.24 |
| ≥12 | 81.8 | 65.7 | 67.2 | 2.39 | 0.28 |
| ≥13 | 81.8 | 71.8 | 72.8 | 2.90 | 0.25 |
| ≥14 | 81.8 | 76.1 | 76.6 | 3.42 | 0.24 |
| ≥15 | 77.3 | 80.3 | 80.0 | 3.92 | 0.28 |
| ≥16 | 59.1 | 85.9 | 83.4 | 4.20 | 0.48 |
| ≥17 | 59.1 | 89.2 | 86.4 | 5.47 | 0.46 |
| ≥18 | 54.6 | 90.1 | 86.8 | 5.53 | 0.50 |
| ≥19 | 50.0 | 92.5 | 88.5 | 6.66 | 0.54 |
| ≥20 | 31.8 | 94.4 | 88.5 | 5.65 | 0.72 |
| ≥21 | 31.8 | 95.3 | 89.4 | 6.78 | 0.72 |
| ≥22 | 31.8 | 96.2 | 90.2 | 8.47 | 0.71 |
| ≥23 | 27.3 | 97.2 | 90.6 | 9.68 | 0.75 |
| ≥24 | 27.3 | 97.6 | 91.1 | 11.62 | 0.74 |
| ≥25 | 27.3 | 98.6 | 91.9 | 19.36 | 0.74 |
| ≥26 | 22.7 | 98.6 | 91.5 | 16.14 | 0.78 |
| ≥27 | 22.7 | 99.1 | 91.9 | 24.20 | 0.78 |
| ≥28 | 13.6 | 99.1 | 91.1 | 14.52 | 0.87 |
| ≥29 | 9.1 | 99.1 | 90.6 | 9.68 | 0.92 |
| ≥32 | 9.1 | 99.5 | 91.1 | 19.36 | 0.91 |
| ≥40 | 4.6 | 99.5 | 90.6 | 9.68 | 0.96 |
| ≥49 | 4.6 | 100.0 | 91.1 | - | 0.96 |
| >49 | 0.0 | 100.0 | 90.6 | - | 1.00 |
