# Supplementary material for: Validation of the Refugee Health Screener-15 for the assessment of perinatal depression among Karen and Burmese women on the Thai-Myanmar border
Source: PLoS One. 2018 May 21;13(5):e0197403. doi: 10.1371/journal.pone.0197403 (PMC5962314; doi:10.1371/journal.pone.0197403)
Supplement: S2 Table — (DOCX) [file pone.0197403.s002.docx]

**S2 Table.** Sensitivity, specificity, likelihood ratios and proportion correctly classified using the Burmese RHS-15 distress thermometer (n=232)

| **RHS-15 cut-off** | **Sensitivity** (%) | **Specificity** (%) | **Correctly classified** (%) | **Positive likelihood ratio** | **Negative likelihood ratio** |
| --- | --- | --- | --- | --- | --- |
| ≥0 | 100.0 | 0.0 | 9.5 | 1.00 | - |
| ≥1 | 100.0 | 17.1 | 25.0 | 1.21 | 0.00 |
| ≥2 | 95.5 | 34.8 | 40.5 | 1.46 | 0.13 |
| ≥3 | 90.9 | 50.5 | 54.3 | 1.84 | 0.18 |
| ≥4 | 77.3 | 66.7 | 68.5 | 2.39 | 0.34 |
| ≥5 | 59.1 | 77.6 | 75.9 | 2.64 | 0.53 |
| ≥6 | 40.9 | 93.3 | 88.4 | 6.14 | 0.63 |
| ≥7 | 36.4 | 95.2 | 89.7 | 7.64 | 0.67 |
| ≥8 | 18.2 | 96.2 | 88.8 | 4.77 | 0.85 |
| ≥9 | 9.1 | 97.6 | 89.2 | 3.82 | 0.93 |
| ≥10 | 4.6 | 99.1 | 90.1 | 4.77 | 0.96 |
| >10 | 0.0 | 100.0 | 90.5 | - | 1.00 |
