# Supplementary material for: Validation of the Refugee Health Screener-15 for the assessment of perinatal depression among Karen and Burmese women on the Thai-Myanmar border
Source: PLoS One. 2018 May 21;13(5):e0197403. doi: 10.1371/journal.pone.0197403 (PMC5962314; doi:10.1371/journal.pone.0197403)
Supplement: S3 Table — (DOCX) [file pone.0197403.s003.docx]

**S3 Table.** Sensitivity, specificity, likelihood ratios and proportion correctly classified using the Sgaw Karen RHS-15 items 1-14 (n=275)

| **RHS-15 cut-off** | **Sensitivity** (%) | **Specificity** (%) | **Correctly classified** (%) | **Positive likelihood ratio** | **Negative likelihood ratio** |
| --- | --- | --- | --- | --- | --- |
| ≥0 | 100 | 0.0 | 6.2 | 1.00 | - |
| ≥1 | 100 | 1.6 | 7.6 | 1.01 | 0.00 |
| ≥2 | 100 | 3.1 | 9.1 | 1.03 | 0.00 |
| ≥3 | 100 | 13.2 | 18.6 | 1.15 | 0.00 |
| ≥4 | 100 | 14.3 | 19.6 | 1.137 | 0.00 |
| ≥5 | 100 | 19.8 | 24.7 | 1.25 | 0.00 |
| ≥6 | 100 | 26.0 | 30.6 | 1.35 | 0.00 |
| ≥7 | 100 | 36.8 | 40.7 | 1.58 | 0.00 |
| ≥8 | 100 | 40.7 | 44.4 | 1.69 | 0.00 |
| ≥9 | 100 | 46.9 | 50.2 | 1.88 | 0.00 |
| ≥10 | 100 | 54.7 | 57.5 | 2.21 | 0.00 |
| ≥11 | 94.1 | 62.8 | 64.7 | 2.53 | 0.09 |
| ≥12 | 94.1 | 68.6 | 70.2 | 3.00 | 0.09 |
| ≥13 | 88.2 | 72.9 | 73.8 | 3.25 | 0.16 |
| ≥14 | 88.2 | 76.4 | 77.1 | 3.73 | 0.15 |
| ≥15 | 88.2 | 81.0 | 81.5 | 4.65 | 0.15 |
| ≥16 | 82.4 | 86.4 | 86.2 | 6.07 | 0.20 |
| ≥17 | 76.5 | 87.6 | 86.9 | 6.17 | 0.27 |
| ≥18 | 76.5 | 90.3 | 89.5 | 7.89 | 0.26 |
| ≥19 | 70.6 | 91.1 | 89.8 | 7.92 | 0.32 |
| ≥20 | 70.6 | 92.6 | 91.3 | 9.59 | 0.32 |
| ≥21 | 64.7 | 95.0 | 93.1 | 12.84 | 0.37 |
| ≥22 | 52.9 | 96.5 | 93.8 | 15.18 | 0.49 |
| ≥23 | 47.1 | 97.7 | 94.6 | 20.24 | 0.54 |
| ≥24 | 41.2 | 98.5 | 94.9 | 26.56 | 0.60 |
| ≥25 | 23.5 | 99.2 | 94.6 | 30.35 | 0.77 |
| ≥27 | 17.7 | 99.6 | 94.6 | 45.53 | 0.83 |
| ≥29 | 17.7 | 100.0 | 94.9 | - | 0.82 |
| ≥32 | 11.8 | 100.0 | 94.6 | - | 0.88 |
| ≥38 | 5.9 | 100.0 | 94.2 | - | 0.94 |
| >38 | 0.0 | 100.0 | 93.8 | - | 1.00 |
