# Supplementary material for: Validation of the Refugee Health Screener-15 for the assessment of perinatal depression among Karen and Burmese women on the Thai-Myanmar border
Source: PLoS One. 2018 May 21;13(5):e0197403. doi: 10.1371/journal.pone.0197403 (PMC5962314; doi:10.1371/journal.pone.0197403)
Supplement: S4 Table — (DOCX) [file pone.0197403.s004.docx]

**S4 Table.** Sensitivity, specificity, likelihood ratios and proportion correctly classified using the Sgaw Karen RHS-15 distress thermometer (n=270)

| **RHS-15 cut-off** | **Sensitivity** (%) | **Specificity** (%) | **Correctly classified** (%) | **Positive likelihood ratio** | **Negative likelihood ratio** |
| --- | --- | --- | --- | --- | --- |
| ≥0 | 100.0 | 0.0 | 6.3 | 1.00 | - |
| ≥1 | 94.1 | 21.0 | 25.6 | 1.19 | 0.28 |
| ≥2 | 82.4 | 39.1 | 41.9 | 1.35 | 0.45 |
| ≥3 | 64.7 | 56.1 | 56.7 | 1.47 | 0.63 |
| ≥4 | 52.9 | 69.2 | 68.2 | 1.72 | 0.68 |
| ≥5 | 41.2 | 77.1 | 74.8 | 1.80 | 0.76 |
| ≥6 | 23.5 | 93.3 | 88.9 | 3.50 | 0.82 |
| ≥7 | 23.5 | 96.4 | 91.9 | 6.61 | 0.79 |
| ≥8 | 11.8 | 97.6 | 92.2 | 4.96 | 0.90 |
| ≥9 | 5.9 | 98.0 | 92.2 | 2.98 | 0.96 |
| ≥10 | 5.9 | 98.8 | 93.0 | 4.96 | 0.95 |
| >10 | 0.0 | 100.0 | 93.7 | - | 1.00 |
