# Supplementary material for: Validation of the Refugee Health Screener-15 for the assessment of perinatal depression among Karen and Burmese women on the Thai-Myanmar border
Source: PLoS One. 2018 May 21;13(5):e0197403. doi: 10.1371/journal.pone.0197403 (PMC5962314; doi:10.1371/journal.pone.0197403)
Supplement: S2 Text — (PDF) [file pone.0197403.s006.pdf]

MAHIDOL-OXFORD TROPICAL MEDICINE RESEARCH UNIT
Shoklo Malaria Research Unit

68/30 Baan Tung Road, 63110 Mae Sot Thailand

☎: +66 5554 5021 Fax: +66 5554 5020 E-mail: rose@shoklo-unit.com

21 March 2018

To whom it may concern:

I hereby grant permission for the open-access journal PLOS ONE to publish the map of SMRU clinic sites, including migrant and refugee sites, labelled as Figure 1 in the accompanying manuscript, under the Creative Commons Attribution License (CCAL) CC BY 4.0 (http://creativecommons.org/licenses/by/4.0/). I understand that this license allows unrestricted use and distribution, even commercially, by third parties.

If you need any further information please do not hesitate to contact me.

Sincerely

Dr Rose McGready

Professor of Tropical Maternal and Child Health

Deputy Director SMRU
